# Evaluation of the Relationship of Screw Pullout and Plate Fracutre in Fixation of Mandible Condyle Fractures: A Mechanistic Study

**DOI:** 10.3390/jcm12134380

**Published:** 2023-06-29

**Authors:** Jakub Okulski, Marcin Kozakiewicz, Rafał Zieliński, Michał Krasowski, Bartłomiej Konieczny

**Affiliations:** 1 Department of Maxillofacial Surgery, Medical University of Lodz, 113st Zeromskiego, 90-001 Lodz, Poland; marcin.kozakiewicz@umed.lodz.pl (M.K.); bkost@op.pl (R.Z.); 2 Material Science Laboratory, Medical University of Lodz, 251st Pomorska, 92-213 Lodz, Poland; michal.krasowski@gmail.com (M.K.); bartlomiej.konieczny@umed.lodz.pl (B.K.)

**Keywords:** plate fracture, screw pullout, ORIF, mandibular condyle, mandibualr ramus, surgical treatment, facial trauma

## Abstract

Background: The mandible is the most injured part of the facial skeleton, and 25–40% of mandibular fractures involve the condyle process. The aim of this study is to answer the question of the relationship between screw pullout and/or plate fracture during osteosynthesis. Methods: We tested polyurethane models of mandibles whose condylar process was cut (simulating a fracture) and fused using plates and screws. Results: A total of 672 plates were tested. A total of 25.6% of them were fractured during the test, with most being fractures of the base of the condyle. More screws (81.97%) are pulled out from the ramus than from the condyle—69.15%. Conclusions: The gold standard in the osteosynthesis of condylar fractures is two straight plates. Other than these, there is no one-size-fits-all plate for every type of fracture. Plates fixed with fewer screws (smaller plates used in higher-lying fractures) are more likely to result in screw pullout. On the other hand, in plates fixed with more screws, plate fracture is more common.

## 1. Introduction

### 1.1. General Information

The mandible is the most injured part of the facial skeleton, and 25–40% of mandibular fractures involve the condyle process [1,2]. Due to the high forces transmitted by the mandible, the connecting material may not fulfill its role of immobilizing the fragments.

### 1.2. Surgical Protocol

Surgical treatment of mandibular condyle process fractures using plate osteosynthesis is still standard and gives the best clinical results compared to conservative treatment methods [1,2,3,4]. However, this method of treatment creates clinical difficulties due to adjacent anatomical structures and postoperative complications. The fixation of bone factions in the area of the mandibular condylar process is technically difficult due to displaced small bone fragments, narrow surgical access, the proximity of the joint surface, and the risk of damage to the maxillary artery or retromandibular vein. During the procedure, it is possible to damage the facial nerve branches and traumatize the temporomandibular joint. Moreover, after the procedure, the function may be impaired (laterotrusion, protrusion, pain, malocclusion, deviation of the chin during the opening and closing of the oral cavity), formation of a visible scar [1]; later, loss of stability of the fixing material is a very unpleasant complication of fracture treatment. This can cause the formation of a pseudoarticular joint, bite disorders, pain, skin fistulas [5], facial nerve damage, and chronic inflammation. Fractures of the plate or removal of fixation screws are the most common causes of the above-mentioned problem.

The authors examined 48 types of plates known from filed plate patents, osteosynthesis plate company catalogs, designs proposed in the literature by researchers from around the world, and their own designs (Medical University of Lodz). The aim of this study is to determine the relationship between screw pullout and/or plate fracture during osteosynthesis.

## 2. Materials and Methods

### 2.1. Plates

There are different types and systems of plates for fixation of craniofacial fractures. In the study, we used plates with screw holes with a diameter of 2.0 mm, which we use in our Maxillofacial Surgery Clinic for osteosynthesis mandibular fractures. On the other hand, we use thinner plates and smaller screws, most often the 1.5 mm system, to join, for example, fractures of the jaws, zygomatic bones, and orbital frames.

We found 48 plate designs for mandibular fracture osteosynthesis. The 48 plates were designed in CAD software, system 2.0. Then, from a sheet of certified medical grade titanium alloy (grade 23, thickness 1 mm), the plates were laser-cut.

### 2.2. Mandibles

Standardized, homogenous, polyurethane mandible models were purchased. The results of biomechanical tests are dependent on the difference in density and bone modulus of elasticity [6,7]. The literature proves that polyurethane mandibles are the material of choice in orthopedic implant tests, especially fractures, which is confirmed by the American Society for Testing and Materials [8,9]. The most natural test material seems to be cadaver bones. Unfortunately, they differ, so the results of biomechanical tests would not be standardized [7]. Still, another option would be to print the mandibles in 3D printers, which would allow the reconstruction of the compact bone and spongy bone. However, due to the need to standardize the models (e.g., so that they are not too rigid compared to human mandibles), the high production costs (well beyond the authors’ financial means), and the presumably long production time, this option was rejected, too. The homogeneous polyurethane material has properties comparable to those of human cancellous bones and is widely used as an ideal medium to mimic human cancellous bones. Polyurethane mandibles (Sawbones, Vashon, WA, USA: density 0.16 g/cc, compression modulus 58 Mpa) were used as models for strength tests [10,11,12,13].

Each condylar process was cut once to simulate one of the three possible fractures (basal fracture, low-neck fracture, and high-neck fracture) in accordance with Kozakiewicz’s classification [14]. In sequence, 1.5 mm diameter calibrated drill, positioned perpendicularly to the surface of the plate, was used to drill holes for screws. Condylar (proximal) and ramus (distal) fracture segments were fixed with a plate, which was screwed, with the same 6 mm long certified titanium self-tapping screws of the 2.0 system. Basal fracture was set and fixed with 48 types of plate, low-neck with 34 types of plate, and high-neck with 14 types of plate (each mentioned fracture underwent seven repetitions for each type of plate).

### 2.3. Simulation Set

**Figure 1:**
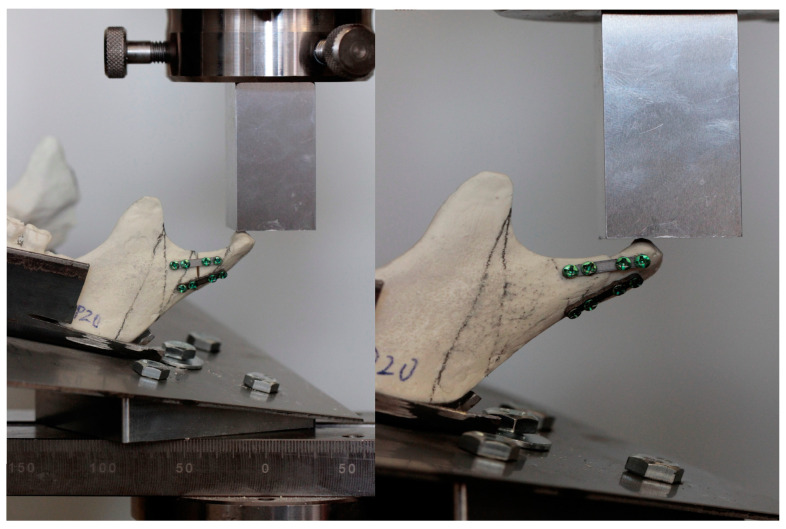
Mandible model during testing.

**Figure 2:**
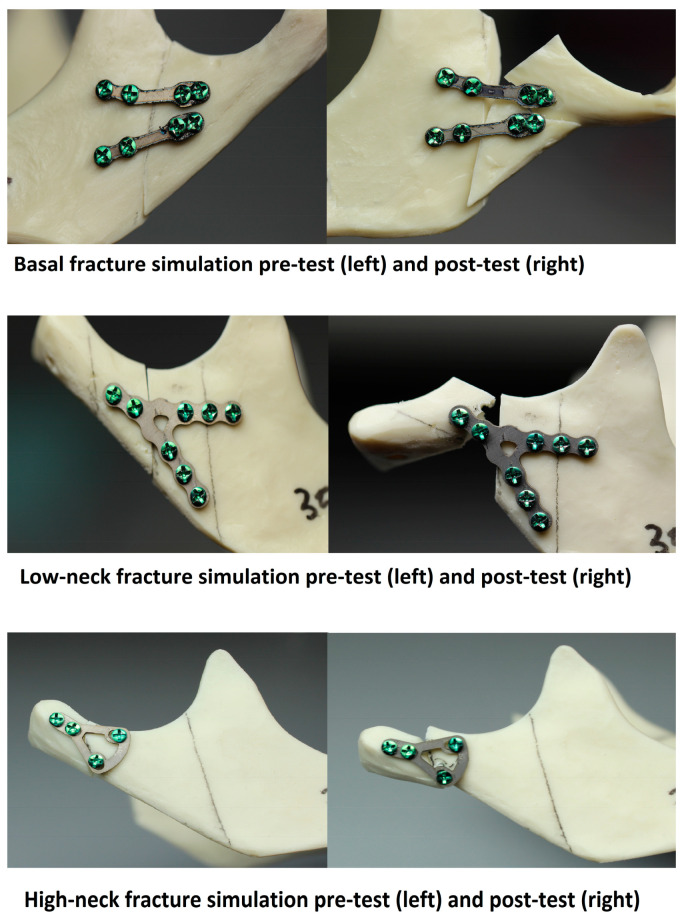
Mandible models before and after testing.

Forces of the temporomandibular joint were simulated according to the literature [15,16,17,18]. We used a truncated cuboid as follows at 15° inferior in the sagittal plane and 10° lateral in the coronal plane. Mandibles were solidly stabilized by screws on the individual base plate [15,16,17]. The plate was 1 mm thick, made with stainless steel, and was screwed on 70 mm × 60 mm tilted block with four M6 holes for stabilization with bolts. In this construction, forces were generated on a basal condyle upward, forward, and medially (Figure 1 and Figure 2) according to the path of stress on the condylar process along the bone beams: along the posterior edge of the mandibular branch and along the mandibular notch. In this way, they replicate as naturally as possible the muscle forces acting on the condylar process.

**Figure 3:**
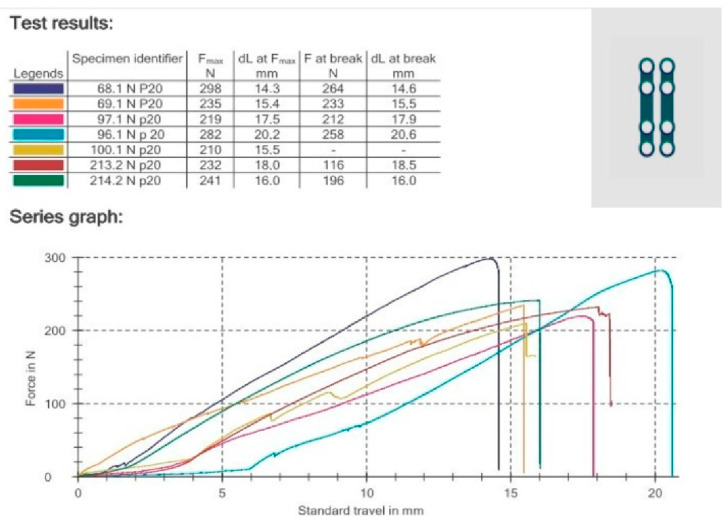
Graph from testXpert II V3.31.

All strength tests were performed using a Zwick Roell Z020 universal strenght machine (Zwick-Roell, Ulm, Germany). The loaded force was 1 N, and the velocity of the piston was 1 mm per minute. All compression forces were pointed to the condyle. Instron software (testXpert II V3.31, Zwick Roell, Ulm, Germany) recorded the relationship between the applied force and displacement of the faction, load for permanent deformation, and maximum load at fracture, Figure 3. In addition, the number of pullout screws and the number of broken plates were recorded. The maximum force needed for a 1 mm condylar displacement in the entire group was 15.72 N.

### 2.4. Statistical Analysis

The results of the research for a given type of fracture and a specific plate were presented in the form of a diagram of the displacement of the fractures as a function of the applied force. The data were then compiled statistically. An analysis of the insert construction was carried out, including the number of post-test anchoring screws and broken plates. Detected relationships were assumed to be statistically significant when *p* < 0.05. Statgraphics Centurion version 18.1.12 (StatPoint Technologies, Warrenton, VA, USA) was used for statistical analyses.

## 3. Results

**Figure 4:**
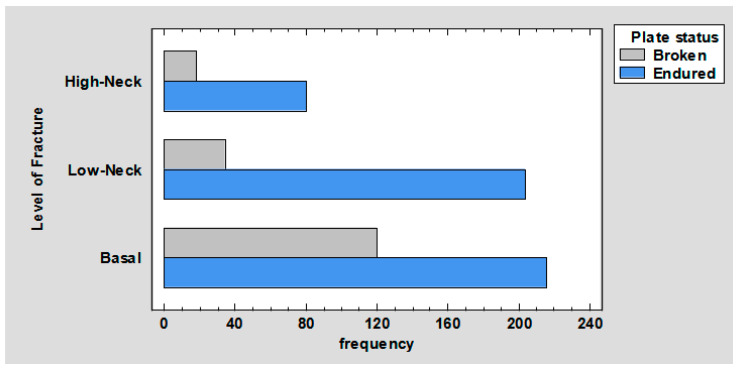
Barchart for level of fracture by plate status.

A total of 672 plates were tested. Of these, 25.6% were broken during testing. This was 17.86% in base fractures (336 plates), 5.06% in low-neck fractures (238 plates), and 2.68% in high-neck fractures (98 plates), *p* < 0.01 (Figure 4).

**Figure 5:**
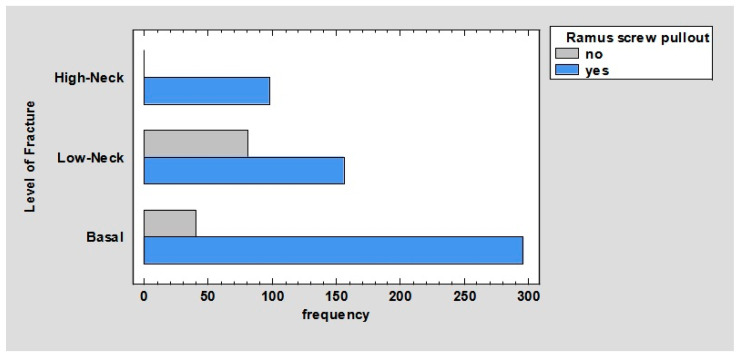
Barchart for level of fracture by ramus screw pullout.

Comparing the number of pullout screws in the ramus according to the fracture, we obtained the following results. In base fractures, 44.11% of the screws fell out; in low-neck fractures, 23.25% of the screws fell out; and in high-neck fractures, all of the screws fell out, *p* < 0.01 (Figure 5).

**Figure 6:**
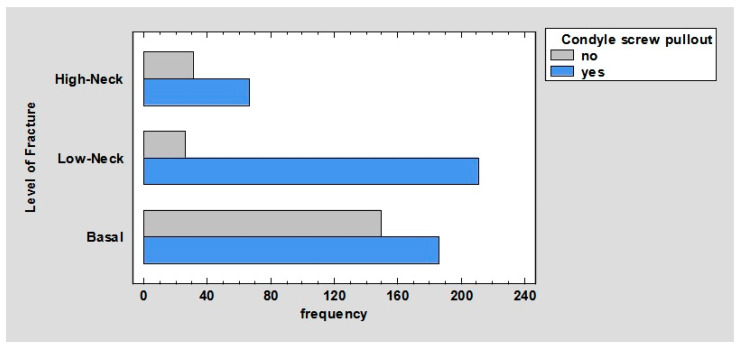
Barchart for level of fracture by condyle screw pullout.

Comparing the number of pullout screws in condyle according to fracture, we obtained the following results. In base fractures, 27.72% of the screws fell out; in low-neck fractures, 31.45% of the screws fell out; and in high-neck fractures, 9.99% of the screws fell out, *p* < 0.01 (Figure 6).

No relationship was observed between plate status (broken or endured) by ramus screw pullout and plate status by condyle screw pullout.

**Figure 7:**
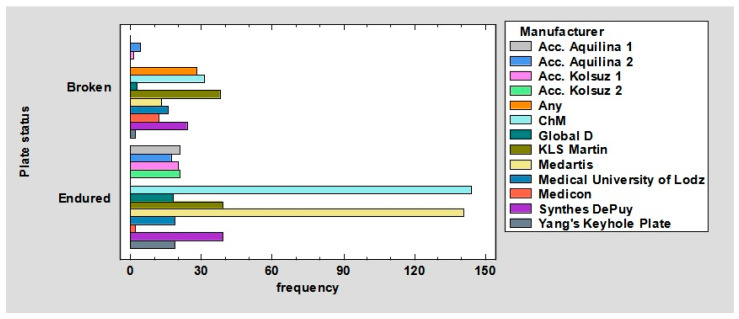
Barchart for plate status by manufacturer.

The relationship between the plate status and the plate’s manufacturer/inventor was observed. Acc. Aquilina 1 plates—broken: 0%, endured: 3.13%. Acc. Aquilina 2 plates—broken: 0.6%, endured: 2.53%. Acc. Kolsuz 1 plates—broken: 0.15%, endured: 2.98%. Acc. Kolsuz 2 plates—broken: 0%, endured: 3.13%. Plates of any manufacturer—broken: 4.17%, endured: 0%. ChM plates—broken: 4.61%, endured: 21.43%. Global D plates—broken: 0.45%, endured: 2.68%. KLS Martin plates—broken: 5.65%, endured: 5.8%. Medartis plates—broken: 1.93%, endured: 20.98%. Medical Univeristy of Lodz plates—broken: 2.38%, endured: 2.83%. Medicon plates—broken: 1.79%, endured: 0.3%. Synthes DePuy plates—broken: 3.57%, endured: 2.83%. Yang’s Keyhole plates—broken: 0.3%, endured: 2.83%; *p* < 0.01 (Figure 7).

**Figure 8:**
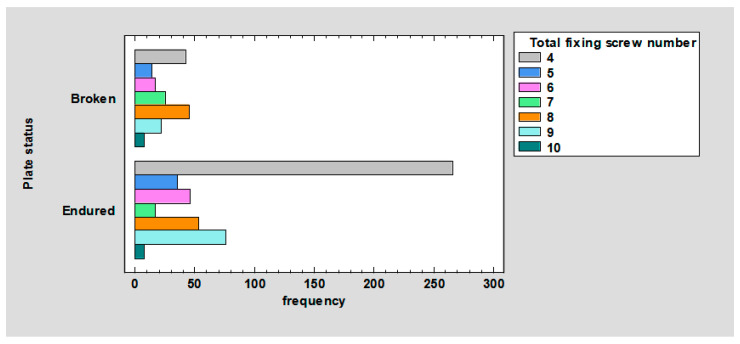
Barchart for plate status by total fixing screw number.

The relationship between plate status and total fixing screw number was observed. For 4 screws—broken: 6.25%, endured: 39.58%. For 5 screws—broken: 2.08%, endured: 5.21%. For 6 screws—broken: 2.53%, endured: 6.85%. For 7 screws—broken: 3.72%, endured: 2.53%. For 8 screws—broken: 6.7%, endured: 7.89%. For 9 screws—broken: 3.27%, endured: 11.31%. For 10 screws—broken: 1.04%, endured: 1.04%; *p* < 0.01 (Figure 8).

**Figure 9:**
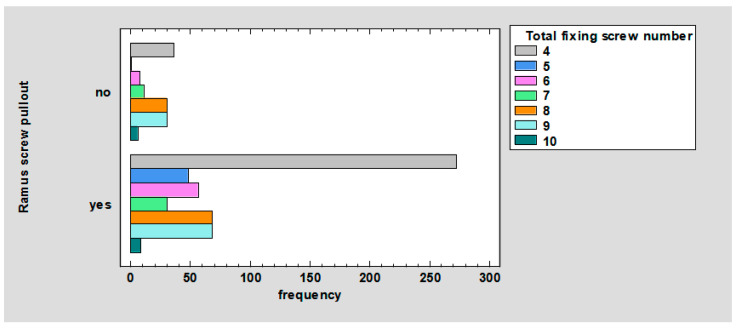
Barchart for ramus screw pullout by total fixing screw number.

The relationship between ramus screw pullout and the total number of fixing screws was observed. For four screws, they did not pull out in 5.37% and pulled out in 40.54%. For five screws, they did not pull out in 0.15% and pulled out in 7.15%. For six screws, they did not pull out in 1.04% and pulled out in 8.35%. For seven screws, they did not pull out in 1.64% and pulled out in 4.47%. For eight screws, they did not pull out in 4.47% and pulled out in 10.13%. For nine screws, they did not pull out in 4.47% and pulled out in 10.13%. For 10 screws, they did not pull out in 0.89% and pulled out in 1.19%; *p* < 0.01 (Figure 9).

**Figure 10:**
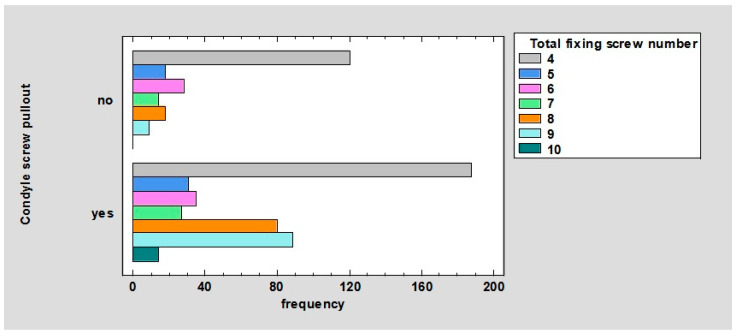
Barchart for condyle screw pullout by total fixing screw number.

The relationship between condyle screw pullout and the total number of fixing screws was observed. For four screws, they did not pull out in 17.88% and pulled out in 28.02%. For five screws, they did not pull out in 2.68% and pulled out in 4.62%. For six screws, they did not pull out in 4.17% and pulled out in 5.22%. For seven screws, they did not pull out in 2.09% and pulled out in 4.02%. For eight screws, they did not pull out in 2.68% and pulled out in 11.92%. For nine screws, they did not pull out in 1.34% and pulled out in 13.26%. For 10 screws, they did not pull out in 0% and pulled out in 2.09%; *p* < 0.01 (Figure 10).

## 4. Discussion

As can be seen from the plate results (Figure 4), plates are most frequently fractured in base fractures, less frequently in low-neck fractures, and least frequently in high-neck fractures. This may be due to the fact that the lower the fracture line is, the higher the forces exerted on the plate, which results in more frequent plate fractures. This relationship is well represented by comparing the force required to displace the fragments by 1 mm, depending on the fracture line, with two straight plates (gold standard). In base fractures, these forces are 15.2 ± 2.69 N/mm; in low-neck fractures, 15.23 ± 3.53 N/mm; and in high-neck fractures, 14.02 ± 1.24 N/mm. The above may also be due to the fact that smaller plates, which are mostly used in more localized fractures, were also tested in base fractures and were not able to cope with the forces acting in base fractures.

It is worth mentioning in the charts (Figure 5 and Figure 6), overall, more screws (81.97%) are pulled out in the distal fracture than in the proximal fracture—69.15%. In the mandibular ramus, all screws are pulled out in high-neck fractures. This may be due to the fact that in these fractures, the plate in the distal fracture is usually fixed with only two screws; hence, it follows that a significantly higher force is exerted on a single screw than in comparison with other fractures where these screws are most often placed. In addition, in these fractures, the thickness of the bone that is available to anchor the screw is less than in fractures whose line passes closer to the angle of the mandible. In addition, the location of the holes close to the fracture line and the low height of the mandible in high-neck fractures can lead to a situation in which the winding of the holes weakens the condylar process and can lead to its fracture in the line of screw insertion.

We used one type of 2.0 × 6.0 mm screws because, in our clinical experience, these are the most optimal screws. The 8 mm screws are usually too long because the condylar process in the frontal plane is not thick. On the other hand, 4 mm screws, after subtracting 1 mm of plate thickness, anchor in the bone over a short length and often do not have sufficient stabilization. It is worth inserting 4 mm screws if there is a suspicion that the lower alveolar nerve canal may run in the place where the plate hole falls out.

One possible solution is to use a different type of screw. According to Gustafson et al. [19], screws with a tapered design are more durable than those with a fixed diameter, as well as screws with a fixed pitch than those with a variable pitch. The screws used in the study were fixed-diameter, fixed-pitch screws. In addition, a smaller bed can be drilled under a tapered screw, thereby weakening the bone less at the screw insertion site.

Another solution is to use self-drilling screws instead of self-tapping screws, which, according to Kozakiewicz et al. [20] and Kumar et al. [21], provide a more stable anchorage in bone. They are characterized by a higher force required to pull them out and a more favorable distribution of stresses and deformations at the bone–screw interface. In addition, in the work of Kozakiewicz et al. [20], longer screws and those with larger diameters required a higher force needed to pull the screws out.

Longer screws allow bicortical anchoring, which intuitively seems to be a very good solution. However, according to Joshi et al. [22], monocortical anchorage provides sufficient stabilization of mandibular fractures from the lingual side and is as reliable as bicortical anchorage.

Another reason for screw pullout may be because the mandibular models we tested are manufactured from a single type of structure that mimics spongy bone. What is missing is cortical bone, which is harder and in which the anchoring of screws would be stronger.

The vast majority of plates have only round holes with a diameter of 2 mm. The exceptions are plates that have an oval hole (usually 1, 2, or 3) for screws with a diameter of 2 mm. This is dictated by the fact that the manufacturer/developer wants to be able to bring the fragments closer together when screwing the insert to make the fracture gap even narrower. There were nine such plates in our study. However, these oval holes were mostly additional, i.e., there were round holes in addition to them. Only one plate had all four oval holes. It seems that the presence of the oval holes may have flowed into the results of a given plate since the screw did not just undergo pulling out of the mandibular model but could still move in the oval hole according to its course as long as the forces acting on the screw coincided with the course of the longer diameter of the oval hole. This is an interesting point worth investigating in future studies.

It can be noted in the chart (Figure 8), the plates were fixed (in order of most frequent occurrence) with 4, 8, 9, 6, 5, 7, and 10 screws. The ratio of the number of broken to surviving plates for four screws is 0.16. Further, respectively, for five, it is 0.40; for six, it is 0.37; for seven, it is 1.47; for eight, it is 0.84; for nine, it is 0.29; for 10, it is 1. Thus, it follows that plates are least likely to break when fastened with 4, 9, 6, 5, 8, 10, and 7 screws. This may be due to the fact that plates fastened with four screws undergo other deformations, such as screw pullout (as confirmed by the results shown in Figure 9 and Figure 10). In contrast, plates fixed with 7, 10, or 8 screws are most likely to fracture because their fixation is the most stable, so the forces are mainly transferred to the plate.

As can be seen in the chart (Figure 9), the breakout of screws from the distal fragment, depending on the total number of anchor screws, ranges from the most frequent for 4, 9, 8, 6, 5, 7, and 10 screws.

For comparison, it is worth referring to the chart (Figure 10); the pullout of screws from the proximal (condylar) fragment, depending on the total number of anchor screws, is the most frequent for 4, 9, 8, 6, 5, 7, and 10 screws. This means that there is no directly proportional correlation between the total number of screws and the strength of the osteosynthesis.

The limitations of this study include the use of polyurethane models of mandibles, which is a uniform structure that mimics spongy bone. It lacks the structure corresponding to compact bone. It should also be noted that all the plates studied were designed by us based on available data because some plates are just concepts found in articles that are not available for purchase; hence, we decided to design all the plates ourselves (rather than purchase them) to make them as comparable as possible. Another limitation is that we used one type of screw to make the tests comparable. The use of, for example, longer and/or self-drilling screws could cause changes in the results. It should be noted that our test is a static-strength test. Under normal conditions, after a mandibular fracture is fused, the patient repeatedly bites while eating; hence, it would be necessary to conduct strength-dynamic tests of repeated rapid and strong force application.

## 5. Conclusions

In summary, in fractures located higher up (high-neck fractures), the forces applied on the plates are lower; hence, smaller plates can be used. In fractures located lower (base fractures), the forces applied on the condyle are higher, and plates as large as possible should be used. In addition, more screws are pulled out in the distal (ramus) fracture, so it is necessary to use plates that are fixed with more screws in the distal than in the proximal (condylar) fracture, e.g., instead of a plate with two holes in the proximal fracture and two holes in the distal fracture, use a plate with two holes in the proximal fracture and three/four/five holes in the distal fracture. The most stable osteosynthesis is found with plates fixated with a total of 5, 7, and 10 screws, although the risk of plate fracture increases with such plates.

## Data Availability

The data presented in this study are available on request from the corresponding author. The data are not publicly available due to an ongoing multicentre project.
